# On Specific Gravity

**Published:** 1818-10

**Authors:** Nicholas Littleton


*For the London Medical and Physical Journal.*

### *On Specific Gravity;* by Nicholas Littleton, Esq.

MORE than two thousand years ago the idea occurred to Archimedes that metals might vary in their density; which led to the discovery of the method of ascertaining the specific gravity of metals, and consequently of all solid and insoluble bodies. Liquid bodies varying so little in their specific gravity, that one common liquid is not sensibly heavier than another, it is probable that the specific gravity of liquids was not discovered at such an early period. And, as the different gases are but recently known, so the specific gravity of these must also have been but lately determined. In all these bodies, whether solid and insoluble, liquids or gases, it may be remarked, that a difference in their specific gravity was no sooner guessed at, than a means of ascertaining it immediately followed.

The methods of ascertaining the gravity were either by observing what weight of water was displaced by certain known weights of the different solids as by weighing in air and water, or it was effected by weighing known bulks of solid, fluid, or aerial substances. More recently the ascertainment of the gravities of fluids has been facilitated by the invention of the hydrometer, and of solids by the gravometer, invented by Mr. Nicholson. So far has this branch of hydrostatics been advanced: the inventive power of the human mind always keeping pace in the ascertainment of facts, with the wandering idea feasting on supposition and guess-work. But, nevertheless, the work is as yet unfinished. How can the specific gravity of such powders as vary but little in their gravity from water, be ascertained? How can the gravity of solid but soluble substances be ascertained? As to the former, Van Swieten even asks, Whether solid hodies, when reduced to an impalpable powder, have not their specific gravity lessened? Mr. John Hunter judges of the specific gravity of the lymph and red globules merely by the suddenness of their sinking in water. Dr. Pearson found it difficult to ascertain the specific gravity of James's powder; and, from not knowing how to ascertain its gravity accurately, he has left his analysis somewhat imperfect.* Also, in popular works on chemistry, either the specific gravity of such powders as carbonate of magnesia, &c. are not mentioned, or, what is much worse, such gravities are stated so as to appear insulting to the understanding. In Dr. Duncan's Dispensatory, the gravity of carbonate of magnesia is said to be

---

* Dr. Pearson, in his analysis of James's powder, mentions the turbid colour caused by the precipitation of phosphate of silver. This turbid or yellowish colour of the phosphate renders it indispensably necessary, for the detection of arsenic, to analyse the precipitate caused by the ammoniaco-nitrate of silver, since the yellowness of precipitates cannot afford sufficient evidence of the presence of arsenic. I have found that the ammoniaco-nitrate of silver causes a muddy yellowness, when dropped into fluids ejected from the stomach by natural vomiting; and, by dropping some solution of arsenic into another portion of the same vomited fluids, no perceptible difference in the colour of the precipitate caused by the argentum nitratum was observed. Chemists, who lay so much stress on the yellowness of the precipitate alone, should recollect that their experiments are not to be performed, as in their laboratories, on a transparent colourless fluid as water, but that they must always expect to find the contents of the stomach more or less coloured. The fluids which I have examined were reddish, yellowish, or greenish; in such fluids, colourless precipitates may appear yellowish, and blue precipitates green. The proposal of observing the colour of the precipitate on white paper seems also, for the same reasons, equally inconclusive: therefore, an analysis of the precipitate, so easily effected, is the only certain test of the presence of arsenic.

0·2941,—not one-third the weight of water! Who does not know that it sinks in water? As to soluble bodies, whose gravity, on account of their great solubility, is not accurately ascertainable by the usual methods, to those the same remarks apply; and, with regard to soluble bodies generally, great incongruity prevails in the results of different experimentalists.

So far my knowledge extends as to what is already known on the present subject, and hence this communication may deserve notice. If, however, the method here adopted be already in use, or commonly known, inserting this letter in your Journal must be unnecessary. In the following simple manner, the first question as to the gravity of light powders is answered:—

1st. The weight of a bottle and cork was found to be.......................... 792 grains

2d. The bottle filled with water weighed...... 1599—792=807

3d. Ditto, nearly filled with the carbonate of magnesia .......................... 865—792= 73

4th. The same bottle still containing the magnesia and filled with water.............. 1605—792=813

813—73=740, the quantity of water required to fill the bottle containing the magnesia. Now, as the bottle would contain 807 grains of water, therefore 807—740=67, the bulk of water occupied by 73 grains of the carbonate of magnesia.

As 67 : 73 :: 1·000 : 1·089, the specific gravity of carb. magnes.

After the same manner, the specific gravity of fibrine (or the buffy coat) was found to be ................ 1·069

And the bottom of the same coagulum, which contains the most red globules ........................... 1·084

In this method, care is required to have the bottle filled exactly to the same height, and to have all the bubbles of air which may be entangled in the powder expelled.

The specific gravity of bodies, however soluble, is ascertained on a similar principle:—

1. The bottle used before, when filled with a saturated solution of subcarb. potass..... 1982—792=1190

2. Ditto, partly filled with dry subcarbonate of potash weighed.................. 1598—792= 806

3. Ditto, ditto, and filled with the saturated solution .......................... 2247—792=1455

As 807 : 1190 :: 1·000 : 1·4745, the gravity of a saturated solution of subcarb. potassæ, 1455—806=649, the quantity of solution in the third weighing 1190—649=541, the bulk of solution occupied by 806 grains of the subcarbonate.

As 541 : 806 :: 1·4745 : 2·1967, the specific gravity of subcarb. potassæ.

Still, more care is required in the operation for soluble bodies than for powders. In soluble bodies, attention must be paid to the temperature of the solution; for, if used soon after making, the result of the experiment will be inaccurate, as during the solution of salts much free caloric becomes latent, and therefore the solution, from its coldness, is of an increased gravity,—is more condensed. The condensation, however, is not entirely dependent on the temperature. This was first detected by finding that the mean gravity of the salt and of the water, in some solutions, was less than the gravity of the solution itself; and it was found also, that a solution, when raised to the temperature of the water used in the solution, was of less bulk than both the water and salt mixed together before solution. This condensation, independent of temperature, shows that solution does not arise merely from a mechanical separation of the particles of bodies, but from a chemical affinity.

There is only one class of bodies whose gravity is not ascertainable by any method yet mentioned: viz. such powders as are lighter than water. These must, on account of their levity, be weighed together with spirit or æther, instead of water. Thus, any substance presented to us, and in whatever form, can easily have its specific gravity ascertained.

Now, as darkness* itself is a reflector of light, so it is not improbable, but that even from a mind of darkness some light may be elicited, and such is the hope of your communicant.

*Padstow; July*, 1818.

---

*For the London Medical and Physical Journal.*

### *History of the Excision of a Portion of the Ribs and Pleura;* by M. Richerand, Principal Surgeon of the Hospital St. Louis.

[From our Correspondent at Paris.]

M. MICHELLEAU, of Nemours, for the space of three years, laboured under a cancerous tumour in the region of the heart; the extirpation of which was performed

* When we look from one place through a transparent medium, into another where there is less light, we may always observe, that the darker place is a reflector. Therefore deep clear water reflects; and hence it is, when we are inattentive to the laws of reflexion in examining the eye, we cannot see the crystalline lens, but only our own image. 4

